# Puerperal Convulsions Treated by the Hypodermic Use of Morphine

**Published:** 1875-05

**Authors:** John D. McGill

**Affiliations:** Surgeon to St. Francis Hospital, Jersey City, New Jersey


### PUERPERAL CONVULSIONS TREATED BY THE HYPODERMIC USE OF MORPHINE.

BY JOHN D. McGILL, M. D.,

*Surgeon to St. Francis Hospital, Jersey City, New Jersey.*

On the evening of September 25th, 1873, I was called to attend, in labor, Mrs. T——, a primipara, seventeen years of age. Although engaged some months previously, I had never visited or prescribed for the patient. Upon my arrival, I found that Mrs. T—— had been in labor four hours, and appeared to be progressing favorably but rather slowly. The os uteri was dilated to about the size of a penny, and somewhat rigid, the child presenting by the vertex-left occipito iliac position. After waiting some time and finding the labor progressing very slowly, I left, with instructions to send for me when the pains became more urgent. At 5.30 the next morning was sent for, and found the patient in the second stage of labor, the head well down in the pelvis. Watching her closely, I noticed that she tossed her arms about, wept passionately, and was very restless generally. This condition was followed by nervous twitchings, especially of the facial muscles, and trembling of the right arm, and the pulse became quickened from eighty-four to one hundred pulsations per minute. These symptoms excited my apprehension, and, being questioned, she stated that she had not passed her water for over twelve hours, and that for the last few days her legs and face had been very much swollen. A catheter was immediately introduced and two or three ounces of dark colored urine drawn, which, upon examination, was found highly albuminous. A few minutes later, the head of the child resting on the perineum, the patient was seized with a violent convulsion lasting eight minutes. Pressure upon the carotids failed to have the slightest controlling effect. Immediately I sent for my forceps and chloroform. Meanwhile another convulsion more violent and prolonged than the first took place, but before it had entirely ceased the forceps were applied and the patient speedily delivered of a living child. The inception of another seizure being noticed, chloroform was exhibited, which appeared to ameliorate but not check a third convulsion. The placenta was extracted without trouble. A semi-comatose condition supervening, with somewhat stertorous breathing, the chloroform was discontinued. The patient continued in a more or less comatose condition for forty minutes, when the restlessness returned, this being speedily followed by a convulsion notwithstanding the use of the anæsthetic. One convulsion followed another, at intervals of fifteen to thirty minutes, a semi-comatose condition, with difficult breathing, in the interim. Chloroform appeared to exercise very little, if any, controlling influence, although it was faithfully applied whenever the nervous phenomena, preliminary to a convulsion, were observed, and its application continued until the spasm ceased.

Two hours or more had now elapsed since the first convulsion. P. 132, very weak ; t. 103° ; skin dry ; resp's, ten per minute.—Chloroform failing in its desired effect, Norwood's tinct. verat. virid. gtts. v. combined with grs. xv. potass. brom. were given every half hour, increasing the dose of veratrum one drop at each exhibition. After the fourth dose, there being no cessation in the frequency of the convulsions, nausea was induced, with subsequent copious vomiting, and the patient became semi-conscious. Pulse, 132 per minute, weaker and smaller than before ; t. 102½° ; resp. ten per minute. The patient soon relapsing into her previous condition—semi-coma with convulsions—veratrum was again employed, gtts. iij., every hour, without the bromide, but with no appreciable effect. This treatment, with a stimulating injection per rectum, was continued four hours without favorable results.

Ten hours had now elapsed, and no amelioration in the condition of the patient was perceptible. She appeared to be sinking fast. Finding that the means ordinarily used failed to control the seizures, and it being very apparent that, unless they should be speedily checked, the patient would succumb, I determined to try the hypodermic injection of morphia advocated by Professor Loomis, of New York City, in cases of convulsions from uræmic poisoning. The patient was in the restless state preceding a seizure. Ten minims of Magendie's solution were injected in the arm, and the effect watched for with much interest. After a few minutes, the restlessness and muscular twitchings subsided, the breathing became more natural, and the threatened attack was undoubtedly aborted. An hour later she was sleeping quietly ; skin moist ; pulse, 120 ; tempature, 102°. Three hours after, another convulsion ensued, not, however, a very severe one. Fifteen minims of sol. magend. were this time injected, and she soon fell off into a quiet sleep, perspiring quite freely. The catheter was introduced and eight ounces of slightly albuminous urine extracted. From this time the patient had no recurrence of the convulsions; the albumen completely disappeared from the urine by the next day; the pulse, temperature and respirations speedily returned to the normal standard, and she recovered without further incident.

The uræmic phenomena witnessed in this case were probably due to mechanical obstruction to the free elimination of urea, caused by the pressure of the gravid womb upon the renal vessels, as shown by the rapid disappearance of the albumen from the urine when the obstacle to a free secretion of urea was removed. In primiparæ, where the abdominal walls are in a state of rigidity, this is a very common cause of uræmia.

In reference to the use of morphia hypodermically, Prof. Loomis claims (*vide N. Y. Medical Record,* August 1, 1873) that its exhibition is unattended by danger to life to the generality of patients affected by acute uræmia. Its almost uniform effect is to arrest muscular spasm by counteracting the effect of the uræmic poison on the nerve-centres, to establish profuse diaphoresis, to facilitate the action of cathartics and diuretics; in short, that it is, thus administered, a powerful eliminating agent.

